# Expression of Concern: MicroRNA-302c enhances the chemosensitivity of human glioma cells to temozolomide by suppressing P-gp expression

**DOI:** 10.1042/BSR-2019-0421_EOC

**Published:** 2022-11-10

**Keywords:** Chemoresistance, Human glioma, MicroRNA-302c, P-pg, Temozolomide

The Editorial Office has been made aware of potential issues surrounding the scientific validity of this paper, hence has issued an expression of concern to notify readers whilst the Editorial Office investigates. It is noted that similarities exist between the miR-302c mimics panel of Figure 3A and the Figure 3A HCT116 miR-208a-3p inhibitor panel of Wu et al. 2019 (doi: 10.1042/bsr20181598), and between the Figure 2G mimics NC panel and the Figure 2D HCT116 Inhibitor NC panel of the same paper by Wu et al. 2019 (doi: 10.1042/bsr20181598).

